# Molecular Targets and Emerging Therapies for Advanced Gallbladder Cancer

**DOI:** 10.3390/cancers13225671

**Published:** 2021-11-12

**Authors:** Matteo Canale, Manlio Monti, Ilario Giovanni Rapposelli, Paola Ulivi, Francesco Giulio Sullo, Giulia Bartolini, Elisa Tiberi, Giovanni Luca Frassineti

**Affiliations:** 1 Biosciences Laboratory, IRCCS Istituto Romagnolo per lo Studio dei Tumori (IRST) “Dino Amadori”, 47014 Meldola, Italy; matteo.canale@irst.emr.it (M.C.); paola.ulivi@irst.emr.it (P.U.); 2 Department of Medical Oncology, IRCCS Istituto Romagnolo per lo Studio dei Tumori (IRST) “Dino Amadori”, 47014 Meldola, Italy; ilario.rapposelli@irst.emr.it (I.G.R.); francesco.sullo@irst.emr.it (F.G.S.); giulia.bartolini@irst.emr.it (G.B.); elisa.tiberi@irst.emr.it (E.T.); luca.frassineti@irst.emr.it (G.L.F.)

**Keywords:** advanced gallbladder cancer, molecular characterization, targeted therapy, immunotherapy

## Abstract

**Simple Summary:**

Biliary tract cancers (BTCs) are rare tumors with devastating prognosis. Gallbladder cancer (GBC) is the most common BTC, and even though recent advances have been carried out in the field of clinical management, research for molecular targets for precision medicine is proceeding at slow steps. This review discuss the molecular targets highlighted to date, focusing on clinical trials exploring the efficacy of precision medicine and immunotherapeutic compounds for the treatment of advanced GBC. Points of strength and weakness of each molecular biomarker are discussed, designing new landscapes for new therapeutic approaches for this malignancy, and suggesting new roles for cytotoxic agents, to date considered the gold standard for patients’ clinical management.

**Abstract:**

Biliary tract cancers (BTCs), for their low incidence, have been often considered together. Gallbladder cancer (GBC) is the most common biliary tract malignancy, characterized by late diagnosis and poor prognosis, and although it is considered a rare tumor in western countries, other areas of the world show considerable incidence rates. In 2010, results from the large phase III ABC-02 clinical trial on GBC identified the gemcitabine and cisplatin combination as the most effective first-line regimen for both GBC and other BTCs. Since then, various systemic therapies have proven active in BTCs in both first- and second-line settings. Molecular profiling has highlighted important genetic differences between GBC and other BTCs, opening new ways for targeted therapy in advanced disease where standard chemotherapies show marginal benefit. Genome-wide data analysis have shown that GBC molecular landscape offer possible strategies for precision medicine approaches, and a better molecular understanding of the GBC is needed to better stratify patients for treatment. In this review, we discuss the molecular targetable agents for GBC, including the results that emerged by clinical trials exploring new treatment strategies.

## 1. Introduction

Biliary tract cancers (BTCs) comprise a heterogeneous group of malignancies that include gallbladder cancer (GBC), ampulla of Vater cancer (AVC) and cholangiocarcinoma (CCA); the latter is further classified into intrahepatic (iCCA), perihilar (pCCA) and distal (dCCA), while pCCa and dCCA are also referred to extrahepatic cholangiocarcinoma (eCCA). BTC incidence varies among different regions, being higher in Asian than in Western countries [1], accounting for 0.6% of new cancer diagnoses worldwide [2]. In particular, the highest incidence, adjusted to standard world population and reported as age-standardized rate (ASR; per 100,000 person-years), was reported in South Korea (ASR = 3.00), while the lowest one was reported in the United Kingdom (ASR = 0.66) [1]. Moreover, high incidence rates are reported in South American countries and Native North Americans [3]. GBC is the most common among biliary tract malignancies, and the sixth most common cancer of the gastrointestinal tract. [2,4]; moreover, it has an overall 5-year survival rate of about 5%, resulting in the worst prognosis malignancy among BTCs [4,5]. Although all BTCs arise in the biliary epithelium, they show important differences in terms of disease behavior, molecular profiles and sensitivity to therapy [6]. GBC usually does not show histological differences compared to the other BTCs, since >90% are adenocarcinomas, but genomic profiling revealed peculiar genomic alterations in BTCs. Specifically, while for BTC molecular alterations have been mainly found to affect FGF, IDH and PI3KCA pathways, GBC is mainly characterized for HER2 alteration; on the other hand, TP53 and KRAS mutations are common in all these malignancies [7,8]. Many of these alterations are potential druggable targets, and many clinical trials have started to explore this path for treatment of BTC. Here we focus on GBC, and review the current treatment options, the altered molecular pathways and their implications for therapy; in this context, we review ongoing clinical trials and try to outline the possible future development in the targeted therapy of GBC.

## 2. Current Treatment Strategies for Advanced Gallbladder Cancer

### 2.1. First-Line Treatment

Chemotherapy is the actual standard of care for advanced disease, as emerged from the results of ABC-02 trial, a large multicenter phase III study that showed a significant survival advantage for cisplatin plus gemcitabine versus gemcitabine alone: overall survival (OS) 11.7 vs 8.1 months (hazard ratio [HR] 0.64; 95% confidence interval (CI) 0.52–0.80; *p* < 0.001) [9]. This trial included 410 patients with heterogeneous BTCs (25% locally advanced, 75% metastatic), with 36.3% GBC. The subgroup analysis highlighted an improved OS with the combination therapy in patients with GBC (*n* = 149; HR 0.61; 95% CI 0.42–0.89) [9]. More recently, the FUGA-BT/ JCOG1113, a Japanese phase III trial that included 68 GBC patients (39% of enrolled patients), found that gemcitabine plus S-1 (an oral fluoropyrimidine combination) is non-inferior to cisplatin plus gemcitabine, with a median OS of 13.4 months in the cisplatin plus gemcitabine arm versus 15.1 months in the S-1 plus gemcitabine arm (HR 0.945; 90% CI 0.78–1.15; *p* = 0.046) [10]. The authors concluded that gemcitabine plus S-1 should be considered a new standard of care option in advanced BTC; nevertheless, they pointed out a limitation of the trial that included only Japanese patients, thus warranting for further studies to be also performed in the Caucasian population [10]. Interestingly, a Japanese phase III trial (KHBO1401-MITSUBA) showed a slight albeit significant superiority of the gemcitabine/cisplatin/S-1 combination over the standard treatment (cisplatin plus gemcitabine) in advanced BTC, including GBC [11].

Another phase III study specifically designed for unresectable GBC (17.7% stage III, 82.3% stage IV) failed to show the equivalence of a modified gemcitabine + oxaliplatin schedule (mGemOx) compared with the standard gemcitabine + cisplatin (GemCis) regimen, although reporting a numerically better OS with mGemOx [12]. On the other hand, oxaliplatin plus capecitabine (XELOX), to be not inferior to GemOx in a phase III trial including as a first-line option in 30 GBC (26% of enrolled patients) [13]. As a first-line therapy, chemotherapy was also associated with tyrosine-kinase inhibitors in a randomized phase III trial enrolling metastatic BTC (including 47 GBC patients), which showed that the addition of erlotinib to chemotherapy with GemOx did not reach an advantage in progression-free survival (PFS, primary endpoint) or OS, although resulting in a higher objective response rate [14].

New combinations are currently under investigation. SWOG S1815 is a randomized phase III trial comparing gemcitabine-cisplatin-nab-paclitaxel to the standard gemcitabine-cisplatin combination in locally advanced/metastatic BTC including GBC: the primary endpoint is OS, the study is awaiting results [NCT03768414].

PRODIGE38-AMEBICA [NCT02591030] is a French randomized phase II/III trial based on similarities between pancreatic and biliary cancers, that compared a modified 5 fluorouracil plus irinotecan and oxaliplatin (FOLFIRINOX) schedule with gemcitabine plus cisplatin in locally advanced/metastatic BTC, including GBC [15]. The study did not meet the primary endpoint of the phase 2 stage (PFS rate at 6 months) [15].

### 2.2. Second-Line Treatment

Chemotherapy-based regimens represent, to date, the standard of care also in second-line treatment. The randomized phase III trial ABC-06 (162 patients, including 21% of cases of GBC), established the superiority of 5-fluorouracil plus oxaliplatin (FOLFOX) versus active symptom control in the second-line setting, with a median OS of 6.2 and 5.3 months, respectively (HR = 0.69, 95% CI 0.50–0.97; *p* = 0.031) [16]. Even though the benefit in terms of median OS was modest, survival rates at 12 months were 25.9% in the FOLFOX and 11.4% in the ASC arm.

In an effort to identify which patients may benefit from a second-line treatment, an Italian retrospective survey was conducted on data from 300 advanced BTC patients treated in second-line for advanced BTC. Of 300 patients analyzed, 53 (18%) were affected by GBC. Multivariate analysis identified four parameters independently associated with prolonged OS: ECOG performance status 0, CA19.9 lower than median value (≤152 U/mL), ≥6 months PFS in the first-line chemotherapy, and previous surgery on primary tumor [17].

The disappointing results outlined above underscore some of the limits of GBC treatment. First, most clinical trials are non-specific for GBC, due to its rarity. Second, chemotherapy trials do not take into account the recent acquisition from molecular understanding of this disease. Indeed, BTCs have a high rate of druggable molecular alterations, thus they derive a clear clinical benefit from targeted therapy [18]. As a consequence, the improvement of treatment strategies for BTC, including GBC, has to consider the molecular pattern of the disease.

## 3. Molecular Characterization of GBC: The Basis for Precision Oncology

**Figure 1:**
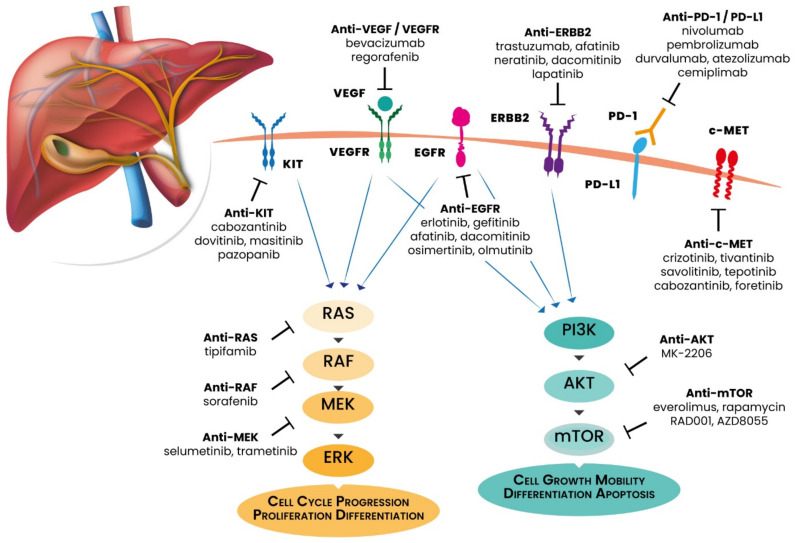
Main actionable gene mutations of gallbladder cancer with relative targeted therapies.

GBC is characterized by peculiar genomic alterations that differ from those observed in the other BTC histologies [19]. Most of these alterations affect cellular signaling pathways involved in cell proliferation and survival, including MAPK/ERK and PI3K/Akt/mTOR pathways and DNA damage repair (DDR) machinery. Moreover, some of these aberrations are clinically relevant and potentially targetable with precision medicine drugs. In this paragraph, we review and discuss the recent advances in the field of GBC molecular characterization, including targetable and non-targetable genomic alterations, highlighted in Figure 1.

In the latest years, with the need to find and validate new targetable alterations for precision medicine, wide genomic characterization through Next-Generation Sequencing (NGS) technologies allowed new approaches for advanced disease treatment in many malignancies, including GBC.

Beyond the molecular differences distinguishing GBC from CCAs, Yang and colleagues highlighted that GBC, as other gastrointestinal malignancies, show molecular differences depending on the ethnicity. Their study revealed that the most frequent genomic alterations in 108 Chinese and 107 GBC patients from the USA were in TP53 (69% and 58%, respectively) and CDKN2A/ B (26% and 25%, respectively), while in the Chinese population other alterations ERBB2 (19%), PIK3CA (17%) and CCNE1 (13%), and in the US population other alterations were SMAD4 (17%), ARID1A (14%) and PIK3CA (14%) ERBB2 (13%) [20].

Some alterations were found in both populations, as high tumor mutation burden (TMB) >10 muts/Mb (17.6% and 17.0%, respectively) and alterations affecting PI3K/mTOR (37% and 33%, respectively), while others were more frequent in the Chinese population (i.e., mutations in ERBB family, 31% vs. 19%, *p* = 0.04) [20]. 

A previous study on 57 tumor-paired GBC patients identified that ERBB family and downstream genes is the most common altered pathway in this malignancy, confirming TP53 as the most commonly mutated gene (47.1%) [21].

These data were later confirmed by Javle and colleagues, who found that ERBB family genes and related pathways could also be affected by somatic copy number alterations (SCNAs) in almost 16% of patients. Moreover, they found that other SCNAs affect MET, FGFR family receptors and chromosome 11q13, accounting for a total of 21% of GBC patients [22].

### 3.1. ERBB Receptors 

ERBB receptors are a four-members family (EGFR, HER2, HER3, HER4) of transmembrane tyrosine kinases that, upon binding of extracellular peptide ligands, undergo conformational changes resulting in homo or hetero-dimerizaion and intracellular cascades activation of RAS-RAF-MEK-ERK, PI3K-AKT-mTOR and PLC-γ1 pathways [23]. EGFR was the first of the family discovered as proto-oncogene, and its expression alterations and mutations has been shown in many cancer cells; moreover, it is a recognized molecular target for precision medicine in molecularly selected lung and colon cancers [24,25]. EGFR signaling seems to play a crucial role also in BTCs tumorigenesis, since EGFR is frequently overexpressed in BTC (100% of ICCs, 52.6% of ECCs, 38.5% of GBCs), and it has been correlated with GBC prognosis [26]. Moreover, EGFR expression has been correlated to gene amplification at high frequency (77%) [27] Specifically in GBC, the overexpression of EGFR has been observed in different studies, although with a high variability among the various reports (expression ranging from 11.3% to 100%) [28]. On the other hand, EGFR mutations have been reported only in about 4% of GBC [21].

Recent studies with the aim of a molecular characterization of GBC identified a subset of GBC patients carrying HER2 alterations (amplification or mutation). In human GBC, HER2 overexpression was found in 9.8–12.8% of cases, and it was highly correlated with gene amplification [27,29,30]. Of note, the report by Roa and colleagues involved 187 cases of GBC: this is the single largest study of HER2 expression in GBC with the commonly accepted criteria of CAP/ASCO (College of American Pathologists/American Society of Clinical Oncology) [30]. On the other hand, a whole-exome sequencing analysis in 157 GBC patients identified mutations of HER2 and HER3 in 7% and 8%, respectively; moreover, this study confirmed the prognostic role of HER/HER3 mutations (median overall survival 8.0 vs 12.3 months in patients without mutation) [31].

Since HER2 gene is a key driver of tumorigenesis, it is also a critical target for therapy. It is a well-established target in breast and gastric cancer, where multiple strategies have been exploited, such as monoclonal antibodies (trastuzumab, pertuzumab), tyrosine kinase inhibitors (lapatinib) and antibody–drug conjugates (trastuzumab emtansine, T-DM1; trastuzumab-deruxtecan) [32,33,34,35,36]. Of note, HER2-targeted therapies have been a major improvement in the management of breast and gastric cancers. Similarly, benefit from trastuzumab, pertuzumab or lapatinib has been reported in HER2-positive GBC [30].

### 3.2. PI3K/AKT/mTOR Pathway

The PI3K/AKT/mTOR pathway activated by ERBB receptors is responsible for stimulating cell proliferation and survival. Most of the molecular alterations affect PI3KCA mutations and amplifications or aberrant expression of AKT and mTOR, as well as somatic mutations affecting the phosphatase tumor suppressor PTEN [37,38]. Molecular alterations affecting this pathway have been found in 4–16% of GBC patients [37,39], while PTEN mutations were found in 4–51% of GBC patients [37,38]. Alterations affecting this pathway have been associated with poorer prognosis in GBC patients [40]. Moreover, PI3K signaling deregulation is an early event in GBC tumorigenesis [41].

PI3K/AKT pathway could also be activated by SPOCK1 (Sparc/osteonectin, cwcv, and kazal-like domains proteoglycan 1), a proteoglycan highly expressed human GBC tissues and associated with patients’ shorter survival. Interestingly, similar roles have been highlighted for fibronectin, a glycoprotein whose levels in human bile fluid increase in malignant biliary diseases, that is able to activate mTOR signaling pathway [42].

### 3.3. MAPK Pathway

The MAPK pathway involve an intricate intracellular phosphorylation cascades and plays a crucial role in cell proliferation and survival, involving key players such as KRAS and BRAF, with a well-known role in carcinogenesis and implications for therapy [43,44].

Even though KRAS mutations are more common in eCCA, they show a frequency of 4–13% in GBC [21,22,45,46,47]. A study on resected GBCs, reported presence of KRAS codon 12 mutations in 16 out of 39 cases (41%), and showed a significantly decreased OS in mutated patients. While BRAF mutations in GBC have been described in a range of 1–5.9% of cases [45], immunohistochemical assay on a large cohort of biliary tract cancers (*n* = 377), including 69 GBC, identified only five cases of V600E mutations, all restricted to the subset of iCCA [48], but this incidence might be underestimated due to use of immunohistochemistry rather than PCR. On the other hand, BRAF gene amplifications have been reported in 5% of a case series of 60 GBCs [49].

Another potential activator of both MAPK/ERK and PI3K/AKT pathways is proto-oncogene c-MET upon the bound of its ligand hepatocyte growth factor (HGF). This receptor is able to induce cell proliferation, resistance to apoptosis and invasion. A study on 113 GBC patients found c-MET overexpression in 39.8% and c-MET gene amplification in 18.3% of the case series; the authors found that c-MET gene amplification was associated with aggressive clinicopathological features and adverse prognosis [50], consistent with other studies that found that c-MET has a negative prognostic role, and in a case series of 35 invasive gallbladder cancers, c-MET was amplificated in 74% of patients [51].

### 3.4. VEGF/VEGFR

VEGF/VEGFR axis guide neo-angiogenesis and neo-lymphangiogenesis, recognized as essential processes in the BTC tumorigenesis [52], as microvessels’ density and vascular endothelial growth factors have been demonstrated to be independent prognostic factors in GBC in two different studies [53,54].

VEGF is a five members family including VEGF-A, VEGF-B, VEGF-C, VEGF-D and the placenta growth factor PLGF. VEGFR family comprises VEGFR1, VEGFR2 and VEGFR3 (tyrosine kinase receptors), and neuropilin-1 (NP-1) and NP-2 (non-tyrosine kinase co-receptors). VEGFR-1 serves as a decoy for the VEGF-A binding to VEGFR-2, leading to activation of PI3K/AKT pathway, while activation of VEGFR-3 leads to both RAS/MAPK/ERK and PI3K/AKT pathway induction [55]. 

Recently, Xu et al. found that VEGF was notably elevated in the serum of patients with GBC and VEGF promoted angiogenesis, cell proliferation and invasion, but inhibited apoptosis in GBC cells [56]. In addition, elevated serum levels and tissue expression of the lymphangiogenic VEGF-C are correlated with tissue expression, lymph node metastases and decreased OS [53]. VEGF gene polymorphisms have also been investigated, and Mishra and colleagues identified c.*237C>T and g.43737830A>G as predictors of disease risk and susceptibility to GBC [57].

### 3.5. DNA Damage Repair (DDR)

The role of DNA damage repair (DDR) pathway, including base excision repair, nucleotide excision repair (NER), double-strand break repair and mismatch repair (MMR) pathways are crucial for repairing or eliminating damaged cells to prevent carcinogenesis [58]. A recent comprehensive genomic profiling performed on a large case series of GBC (*n* = 760) showed that 14.2% of GBC carry a DDR gene alteration, and ATM was the most frequently altered gene (about 50% of all alterations), and, as expected, the authors found that DDR alterations correlated with an higher tumor mutation burden [59]. Another large study including 353 GBC found that BRCA1 and -2 were mutated in 0.3% and 4%, respectively; moreover, a correlation between BRCA mutants and high microsatellite instability (MSI)/deficient MMR was highlighted in the whole BTC case series, suggesting that a subset of BTC patients could benefit from checkpoint targeting [60]. In this subset of patients, recent evidences highlight the possible efficacy of PARP-inhibitors for patients with ATM, BRCA1 [61,62,63].

### 3.6. Programmed Death-1 (PD-1) and PD-Ligand 1 (PD-L1)

In the era of immune checkpoints inhibition for cancer clinical management, the role of programmed death-1 (PD-1) expressed by T cells and its ligand programmed death-ligand 1 (PD-L1) expressed by cancer cells and anti-inflammatory cells have been investigated also in GBC. The principle to inhibit PD-1/PD-L1 binding to re-establish immune effectors against cancer cells and counteract cancer immune escape has reached important results and entered the clinical practice for advanced bladder, melanoma and lung cancers [64]. A study on 66 GBC patients reported that 54% of patients were IHC positive for PD-L1 expression; in particular, 18% and 36% of positivity were found for tumor cells and peritumoral immune stroma, respectively [65]. Later, in a study involving 83 GBC patients, PD-L1 expression on both cancer cells and tumor-infiltrating lymphocytes (TILs) and PD-1 expression on TILs were investigated, and expression levels were found in 15.7%, 13.3% and 51.8%, respectively [59]. Interesting results came from exploration of the relation between immune checkpoints and molecular markers of GBC. Li and colleagues found that PI3K pathway activation mediated by HER-2/HER-3 acquired mutations is able to regulate PD-L1 expression, contributing to cancer immune escape [31]. This result suggests that blocking immune checkpoints could also serve to counteract the effects of activating HER-2 and HER-3 mutations. Moreover, a study involving 203 GBC patients demonstrated a correlation between PD-L1 expression and high TMB [66]. As for other malignancies, the role of PD-1 and PD-L1 expression was investigated in predicting patients’ prognoses, with conflicting results. Two different studies involving 158 and 101 GBC patients, focused on circulating and tissue expression of PD-L1 found that median OS for patients with high protein expression vs patients with low expression were 7.9 vs 14.1 months (*p*  <  0.001) and 50.1 vs 27.8 months (*p* =  0.049), respectively [67,68]. On the other hand, another study did not find correlation between PD-1 and PD-L1 expression in terms of OS [69].

### 3.7. The Hedgehog Pathway

The Hedgehog pathway, which plays a key role in developmental phases, is often reactivated during carcinogenesis, via pathway proteins mutations or over-expression [70]. The first evidence that this pathway is upregulated in GBC was conducted in a case series of 93 patients, finding that Sonic Hedgehog (sHh), its receptor Patched-1 (Ptch-1) and the cytoplasmic effector Gli1 were expressed in 76 (81.7%), 70 (75.3%) and 66 (70.0%) patients, respectively, and were significantly associated to worse patients prognosis [71]. This result was later confirmed by Matsushida and colleagues, who found high levels of sHh, Gli1 and its activator Smo in 37 GBC specimens as compared to healthy gallbladder tissues; moreover, the authors demonstrated that the inhibition of the effector Smo decreases GBC invasiveness through the inhibition of metalloproteinases 2 and 9 (MMP2 and 9) [72].

## 4. Advances in GBC Clinical Management Treatment

Precision medicine is becoming the gold standard in many malignancies, and tumor molecular characterization allowed to discover, target and validate new biomarkers in this scenario. Even though several efforts have been also conducted in GBC, still no biomarkers have been validated and entered clinical practice. In this paragraph, we review and discuss the recent results of clinical trials exploring efficacy of targeted therapies for GBC clinical management.

### 4.1. EGFR Inhibitors

Targeting EGFR is one of the most common strategies in clinical oncology. The main strategy adopted by several randomized phase II trials in BTCs was to combine anti-EGFR monoclonal antibodies (mAbs), i.e., cetuximab or panitumumab, with chemotherapy, in particular gemcitabine plus oxaliplatin. A multicentric phase II trial enrolled 22 GBC patients, with PFS at 4 months as a primary endpoint aimed to evaluate the addiction of cetuximab to GemOx schedule. Even though an observed slight antitumor activity of cetuximab, this did not translate into a survival advantage [73]. The same results were achieved in 14 GBC by Chen and colleagues, together with the evidence that KRAS mutation status cannot be considered a predictive biomarker for EGFR blocking [74]. This result was further confirmed by another phase II study that enrolled 28 KRAS wild-type GBC patients, that used panitumumab in combination with the GemOx regimen [75]. Interestingly, another study reported a higher objective responsa rate (ORR) when adding panitumumab to chemotherapy compared to the addition of the anti-VEGF bevacizumab to the same regimen [76]). A randomized phase III trial that evaluated the association of the TKI erlotinib to a chemotherapy regimen with gemcitabine and oxaliplatin did not result in an increase in PFS (primary endpoint) while highlighting a higher ORR with the combination treatment [14].

Other phase II studies tested anti-EGFR mAb in combination with gemcitabine-based doublet chemotherapy as a first line treatment. Although including GBC and other BTCs and both locally advanced and metastatic settings, these studies showed promising results, with ORR of 31–63% [77,78,79]. 

Morizane did an extensive review on results from phase II trials using targeted therapies (erlotinib, cetuximab, panitumumab ± bevacizumab, sorafenib, cediranib, trametinib and vandetanib) as monotherapy, in combination with cytotoxic agents. Those randomized phase II trial considered GemOx, GemCis therapy or GEM monotherapy to evaluate the additional effect of targeted therapies [19]. Unfortunately, neither EGFR agents nor VEGF agents alone or in combination which each other or in combination with chemotherapy described utility in first line setting.

A meta-analysis by Cai et al. analyzed the results of three randomized phase II and one phase III clinical trials to assess the efficacy and safety of the addition of an anti-EGFR targeted therapy to GemOx. This meta-analysis considered 634 patients with BTC, 146 of which were affected by GBC. The authors concluded that the addition of EGFR-targeted therapy to GemOx resulted in an improvement of both PFS and ORR, but not in a survival advantage. Furthermore, the benefit of anti-EGFR therapy was higher in patients with CCA than in GBC or ampulla of Vater cancer [80]. Another meta-analysis also including studies yet analyzed by the above mentioned one, examined four phase II trials, in which an anti-EGFR mAb (two studies with cetuximab, two with panitumumab) was added to gemcitabine-based first-line chemotherapy (three studies with gemcitabine + oxaliplatin, one with cisplatin + gemcitabine). The meta-analysis concluded that the addition of anti-EGFR mAb did not improve ORR, PFS or OS [81]. 

Some studies have explored the feasibility of targeted therapy alone. For example, Philip et al. reported a phase II study with the TKI erlotinib in 42 patients with advanced BTC, including 16 cases of GBC. The overall confirmed response rate was 8% and the median time to progression (TTP) was 2.6 months; moreover, EGFR expression was not significantly associated with clinical outcome [82]. On the other hand, a phase II trial with lapatinib monotherapy in 17 cases of BTC resulted in a ORR of 0% [83]. There are also experiences of targeted therapy combination: El-Khoueiry et al. reported lack of efficacy when combining erlotinib with the TKI sorafenib (multi-target acting of the VEGF axis) in a phase 2 study including 14 patients with GBC and 20 with CCA. The combination resulted in two unconfirmed PRs (6%) with a median PFS of 2 months (95% CI 2–3), and a median OS of 6 months (95% CI 3–8) [84].

### 4.2. HER2 Blockade

HER2 blockade is another strategy that has been investigated for GBC treatment. Results from the basket trial MyPathway showed a benefit with trastuzumab + pertuzumab in and a cohort of 11 biliary cancer patients with HER2 alteration (eight amplification/overexpression, three mutations) [85].

Pre-clinical in vitro and in vivo evidence showed that pretreatment with gemcitabine/5-fluorouracil enhances trastuzumab cytotoxicity in HER2-negative GBC. Indeed, gemcitabine/5-fluorouracil increased the expression of HER2 and the upregulation of phosphorylated HER2 and AKT, indicating an activation of the HER2/AKT pathway and enhancement of trastuzumab cytotoxicity [86]. These data disclose intriguing perspectives for a sequential treatment strategy even in HER2-negative disease. A retrospective analysis analyzed the outcomes of nine patients with GBC and five with CCA that received anti-HER2 therapy (trastuzumab, lapatinib or pertuzumab). In the GBC group, eight patients had HER2 gene amplification or protein overexpression, one patient carried a HER2 mutation (V777L). In the first group, five responses were observed four partial response (PR) and one complete response (CR) and three stable disease (SD) with HER2-targeted therapy, while the latter experienced a mixed response after lapatinib therapy. The median duration of response was 40 weeks. Interestingly, one patient had developed HER2 amplification after FGFR-directed therapy for FGF3-TACC3 gene fusion [30].

In another study, treatment with lapatinib did not prove efficacy in a subset of 17 BTC patients, including five cases of GBC; of note, the cohort was not selected for HER2 alteration [83].

The SUMMIT phase II basket trial evaluated the activity of the pan-HER TKI neratinib in HER2-mutated cancers, including a cohort of 19 BTCs (42% of which are GBCs) refractory to gemcitabine and platinum-containing regimens. In the BTC cohort, ORR was 10.5%, while clinical benefit rate was 31.6%; considering two PRs and four patients with SD, median PFS was 1.8 months (95% CI, 1.0–3.7). Interestingly, a post-progression biopsy in one patient with GBC revealed loss of HER2 mutation [87]. A pooled analysis of three phase 1 studies with another pan-HER TKI, varlitinib, in combination with platinum-based chemotherapy in 43 BTC patients, 16.3% of whom with GBC, resulted in 10 PRs (27.0%) and 16 SD (43.2%), with a disease control rate (DCR) of 70.3% [88].

Targeting HER2 demonstrated to be a promising strategy using a Trastuzumab biosimilar in a recent study by Jeong et al, that reached a 100% disease control rate (DCR) and a 50% ORR [89].

A new compound, zanidatamab, was tested in a phase I study including 20 BTC patients (11 GBC); results show that the drug is well tolerated (70% of grade 1-2 adverse events), with a median duration of response of 6.6 months (95% CI, 3.2-NR) [90].

An ongoing phase II clinical trial (NCT04183712) aims to assess the feasibility of an approach, based on genomic and proteomic profiling, that includes the combination of the pan-HER TKI afatinib with GemOx in resectable GBC, and patients’ monitoring by circulating tumor DNA (ctDNA). The targeted accrual is 54 GBC patients.

There are currently several ongoing trials aiming to assess the role of HER2-targeted therapies in BTC. Three of these trials (NCT03613168, NCT02992340, NCT02836847), especially as front-line treatment in combination with systemic chemotherapy, while a study for HER2 positive patients with unresectable or recurrent GBC investigating the efficacy of combination of trastuzumab and deruxtecan is currently ongoing in Japan (JMA-IIA00423). With regard to second-line treatment, the TreeTopp (NCT03093870) trial is investigating the efficacy of varlitinib plus capecitabine versus capecitabine plus placebo in patients who have received and failed one prior line of systemic treatment. In the same setting, a phase II trial is currently evaluating trastuzumab plus chemotherapy in previously treated HER2 positive patients (NCT03185988).

A phase II trial, part of the ROAR basket trial, investigated the potential of MEK 1/2 inhibition, and showed activity of the dabrafenib + trametinib combination (BRAF and MEK inhibitors, respectively) in BRAF V600E-mutated BTC, progressed or intolerant to to gemcitabine based-chemotherapy: the cohort included 1 GBC out of 43 cases; ORR in the whole cohort was 51% (in the investigator assessment, 47% in the independent assessment) [91]. Another phase II trial, conducted in Japanese patients with advanced BTC refractory to gemcitabine-based chemotherapy, showed no activity of the MEK inhibitor trametinib in a Japanese cohort (*n* = 20, including 8 cases of GBC) unselected for BRAF status. The trial failed in the primary endpoint (12-week non-progression disease rate), RR was 0% in the investigator assessment, 5% in the independent assessment) [92].

Similar results, non-significant activity as second line treatment for CCA, were obtained in SWOG S1310 study that recruited 44 patients (32% GBC patients). The ORR of trametinib therapy was 10% (95% CI 0–23) vs. 8% (95% CI 0–19) seen in fluoropyrimidine therapy and the mPFS in trametinib therapy was 3.3 months in contrast to 1.4 months in fluoropyrimidine therapy [93]. The trial was stopped because of the lack of response observed in the trametinib arm.

Selumetinib, a second generation of MEK1/2 inhibitor, demonstrated that acceptable tolerability with mPFS and mOS of 3.7 months (95% CI 3.5–4.9 months) and 9.8 months (95% CI 5.97–not available), respectively, in 28 metastatic BTC patients (7 GBC patients) [94].

Interesting results of binimetinib and capecitabine association on RAS/RAF/MEK/ERK mutated BTC patients come from a phase Ib study, that showed a good tolerability and a better tumor response (*p*  =  0.028), PFS (5.4 vs. 3.5 months, *p*  =  0.010) and OS (10.8 vs. 5.9 months, *p*  =  0.160) with respect to wild type patients [95].

MEK 1/2 inhibition brought some interesting evidence, especially for combination strategies; even contrasting, these results need to be better elucidated in further studies, suggesting that this strategy could have a role in the future for GBC treatment. 

### 4.3. PI3K/AKT/mTOR Pathway Inhibitors

As discussed, the PI3K/AKT/mTOR pathway is often altered in GBC. A phase I trial (NCT00949949) explored the maximum tolerated dose (MTD) of mTOR inhibitor everolimus in combination with gemcitabine or gemcitabine plus cisplatin. Ten patients with cholangiocarcinoma or GBCs were included in the full combination arm, and the results showed that six patients had a SD, and four patients experienced a progressive disease [96]. A multi-institutional phase II study of MK-2206 a single-agent targeting AKT, exhibited acceptable tolerability in eight patients with advanced, refractory BTCs [97].

There are phase II studies with everolimus as single treatment for advanced BTCs in first [98] and second line [99] treatment with response rate ranging from 12% in first line to 5.1% in second line and DCR was significantly worse for gall bladder carcinoma compared to other anatomical sites [98].

Data based on very small and explorative case series lead to an uncertain role for these drugs, probably a more extensive molecular characterization could reveal if this pathway could be considered as a driver for GBC, thus helping patients selection.

### 4.4. Mutated TP53 Inhibitors

As all tumors, TP53 is the most mutated gene also in GBC, and targeting mutated p53 has been attempted for this malignancy. Makower et al. performed a phase II trial including 20 patients (15 with mutated p53) and using oncolytic adenovirus ONYX-015 (dl1520, CI1042), a genetically modified adenovirus with a deletion of the E1B gene, thus designed to preferentially duplicate in p53-mutated cells. Of the 19 evaluable patients, 1 of 16 (6.3%) had a PR, 1 of 16 (6.3%) had prolonged disease stabilization (49 weeks), and 8 of 16 (50%) had a >50% reduction in tumor markers [100]. A better understanding at a molecular level of this approach for therapeutic efficacy and safety remain to be clarified.

### 4.5. VEGF/VEGFR Axis Inhibitors

The targeting of VEGF/VEGFR binding has also been explored as a possibility for GBC therapeutics. Small molecules as bevacizumab, lenvatinib and ramucirumab, and TKIs such as vandetinib, sorafenib, sunitinib, apatinib and regorafenib demonstrated clinical activity in many gastrointestinal malignancies, and some results are available for GBC.

Bevacizumab activity in combination with gemcitabine and oxaliplatin for GBC was explored by a single arm phase II trial, demonstrating a 40% ORR and mPFS of 7 months [101]. Other two phase II trials (NCT00356889 and NCT01007552) tested the clinical utility of bevacizumab in combination with erlotinib and gemcitabine and cisplatin, respectively, demonstrating mOS of 9.9 and 10.2 months, respectively [102,103].

Regorafinib was used by a phase II study (NCT02053376) in pre-treated and advanced BTC, leading to a mPFS of 15.6 weeks and a mOS of 31.8 weeks. In this study, 11% of PR and 44% of SD were observed [104].

As a monotherapy, lenvatinib was used in second-line treatment in unresectable BTC patients, demonstrated a DCR of 88% for investigators, and 46% by an independent review, with a mOS of 7.4 months [105].

A multiple inhibitor of RTK, sunutinib, was tested in a phase II trial, with low efficacy for the enrolled BTC patients. In fact, median TTP was 1.7 months, ORR was 8.9% and DCR was 50% [106].

Negative results came from studies investigating vandetanib and ramucirumab as monotherapies (phase II NCT00753675 and phase I NCT02443324), with low responses and survival times [107,108].

VEGF/VEGFR targeting has not led to clear results for clinical benefit. Case control studies are needed to better elucidate the efficacy of such molecules. Moreover, a large panel of small molecules and TKIs has been tested, with different affinities and targets, which efficacy could also be influenced by gene polymorphisms, and to date it remains to be elucidated which could be the best strategy to adopt.

Poly adenosine diphosphate-ribose polymerase inhibitors (PARPis) are emerging therapies for cancer patients carrying germline or somatic alteration in DDR genes. These alterations could also have a prognostic and a predictive role for patients treated with platinum-based therapies [109]. Even though results from clinical trials are still not available (e.g. NCT03878095) for these drugs efficacy in GBC, PARPis developing as monotherapy could be a potential walkable way for GBC, as reviewed by Ricci et al. [110].

### 4.6. Immunotherapy for GBC

PD-1/PD-L1 inhibitors are actually changing the clinical management of many malignancies, especially melanoma and lung cancer.

Nivolumab (anti PD-1) reached important results for GBC treatment in several trials. A phase II trial enrolling 54 pre-treated BTC patients (26% GBC), demonstrated a good tolerability and a 60% DCR in the overall case series with a mPFS 3.98 months (95% CI: 2.33–5.98) and a mOS of 14.22 months (95% CI: 6.64–NA) [111]. A phase I trial (MakotoUeno, JapicCTI-153098) enrolled 30 BTC patients receiving nivolumab as monotherapy, or nivolumab and cisplatin+gemcitabine. The study showed that the median OS were 5.2 months (90% CI, 4.5–8.7) and 15.4 months (90% CI, 11.8–NR), respectively, while the mPFS were 1.4 months (90% CI; 1.4–1.4) and 4.2 months (90% CI, 2.98–5.6), respectively [112].

Immunotherapeutic approaches including combination with anti-angiogenic molecules have also been investigated. Recent results from the phase II multicohort study LEAP-005 highlighted that lenvatinib (anti VEGFR-1-2-3) added to pembrolizumab (anti-PD-1) has a good tolerability, reaching 10% of ORR and 68% of DCR in 31 patients with BTC [113]. A phase II study IMbrave 151 evaluated the role of bevacizumab (anti-VEGF) in combination with atezolizumab (anti-PD-L1) and GemCis as a first-line therapy is currently ongoing [114].

An Asian phase II study uses camrelizumab (anti-PD-1) in combination with FOLFOX or GemOx in untreated population. Data on 43 evaluable patients show an ORR of 7% and a DCR of 67.4%. On the other hand, 57.4% of patients experienced at least a grade 3 treatment-related adverse events [115].

In addition, durvalumab (anti PD-L1) brought interesting evidences, as it tested with or without tremelimumab (anti-CTLA-4) in a phase I trial. Median duration of responses were 9.7 for durvalumab alone and 8.5 months for combination arm, with mOS of 8.1 and 10.1 months, respectively [116].

Even though only preliminary results are available for PD-1/PD-L1 blockade in GBC, to date it seems that this strategy could be the way that could change GBC clinical management. As in treatments for other malignancies, immune checkpoint blockers are emerging as therapy-changing actors, and more clinical trials evaluating the efficacy of these molecules in GBC are warranted. On the other hand, predictive biomarkers are urgently needed for these treatments.

The most representative clinical trials involving advanced gallbladder cancer patients and testing the efficacy of targeted therapies or immunotherapy are resumed in Table 1.

## 5. Conclusions

Personalized medicine for GBC remains a clinical challenge. Mechanisms of carcinogenesis, progression and drug resistance could be better elucidated using a wide molecular characterization, that already provided an emerging scenario of the main altered pathways of the malignancy open the way for new drugs evaluation.

Remaining chemotherapy the gold standard for GBC, the combination of cytotoxic agents with precision medicine compounds or immune checkpoint inhibitors highlighted interesting results that need to be deepened.

In addition, in these cases, molecular wide characterization should be addressed to the discover of predictive and acquired resistance markers, paving the way for new possible treatment strategies for this malignancy. In this context, liquid biopsy is becoming a very useful tool to monitor response to targeted therapies and to investigate the onset of resistance mutations to targeted therapies in many malignancies, and could act a key role also for GBC clinical management.

## Figures and Tables

**Table 1:** Representative ongoing clinical trials involving advanced gallbladder cancer patients and testing the efficacy of targeted therapies or immunotherapy.

Study Code	Cohort	Drug Investigated	Phase	Setting (line)	Drug Target
NCT03260712	BTC and GBC	Pembrolizumab	II	Palliative (I)	PD-1
NCT03111732	BTC and GBC	Pembrolizumab	II	Palliative (II>)	PD-1
NCT02834013	Solid tumors, GBC	nivolumab and ipilimumab	II	Palliative (II>)	PD1 + CTLA-4
NCT03473574	CCA and GBC	durvalumab and tremelimumab	II R	Palliative (I)	PDL1 + CTLA-4
NCT03201458	CCA and GBC	atezolizumab, cobimetinib	II R	Palliative (II>)	PD-L1 + MEK
NCT01308840	BTC and GBC	Panitumumab	II	Palliative (I)	EGFR
NCT01267344	BTC and GBC	Cetuximab	II R	Palliative (I)	EGFR
NCT00478140	BTC and GBC	Trastuzumab	II	Palliative (II>)	HER2
NCT00361231	BTC and GBC	Bevacizumab	II	Palliative (I>)	VEGFR
NCT00356889	BTC and GBC	Bevacizumab, erlotinib	II	Palliative (I)	VEGF + EGFR
NCT02520141	BTC and GBC	Ramucirumab	II	Palliative (I>)	VEGFR
NCT02115542	BTC and GBC	Regorafenib	II	Palliative (III>)	RAF
NCT00919061	BTC and GBC	Sorafenib	II	Palliative (I)	RAF
NCT00238212	BTC and GBC	Sorafenib	II	Palliative (II>)	RAF
NCT00832637	BTC and GBC	Erlotinib	II	Palliative (I)	EGFR
NCT00033462	BTC, GBC and LC	Erlotinib	II	Palliative (II>)	EGFR
NCT01093222	BTC and GBC	Erlotinib, sorafenib	II	Palliative (I)	EGFR + RAF
NCT00350753	BTC and GBC	Erlotinib, bevacizumab	II	Palliative (II>)	EGFR + VEGF
NCT04183712	GBC	Afatinib	II R	Adjuvant	HER/HER EGFR
NCT02992340	BTC and GBC	Varlitinib, cisplatin, gemcitabine	I–II	Palliative (I)	HER2/EGFR
NCT03093870	BRC and GBC	Varlitinib, capecitabine	II–III	Palliative (II)	HER2/EGFR
NCT02151084	BTC and GBC	Selumetinib	II R	Palliative (I)	MEK
NCT01242605	BTC and GBC	Selumetinib	I	Palliative (I)	MEK
NCT02042443	BTC and GBC	Trametinib	II R	Palliative (II>)	MEK
NCT03027284	CCA and GBC	Merestinib	I	Palliative (I)	C-met
NCT02631590	BTC and GBC	Copanlisib	II	Palliative (I)	PI3K
NCT01425879	eCCA and GBC	MK2206	II	Palliative (II>)	Akt
NCT00949949	BTC and GBC	Everolimus	I	Palliative (I)	mTor

Abbreviations: BTC: biliary tract cancer; GBC: gallbladder cancer; LC: liver cancer CCA: cholangiocarcinoma; eCCA: extra-hepatic cholangiocarcinoma; R: randomized.
